# Magnetic Key Hole Technique (MKH) for Multi-Sensorics Local Tests of Soft Magnetic Laminations Under Defined Conditions of Induction

**DOI:** 10.3390/s26103037

**Published:** 2026-05-12

**Authors:** Helmut Pfützner, Georgi Shilyashki, Yusuke Kawamura, Claes Bengtsson, Neofitos Christodoulou, Georgios Christodoulou

**Affiliations:** 1 Magnet Test Labs, ViennaMagnetics GmbH, 1020 Vienna, Austria; 2 Institute of Biomedical Electronics, TU Wien, 1040 Vienna, Austria; 3 Steel Research Laboratories, Nippon Steel Corporation, Chiba 293-8511, Japan; 4 Noratex, Pallini, 153 51 Athens, Greece

**Keywords:** electric steels, amorphous ribbons, losses, magnetostriction, domains, scanning sensors

## Abstract

Inspired by the key hole concept in micro-surgery, we developed a “magnetic key hole concept” for basic studies of localized characteristics of soft magnetic laminations (electric steel, Fe-based amorphous ribbon) under exactly defined conditions of induction *B*(*t*). A material sample of 50 cm length and 10 cm width is magnetized in a novel multi-frequency SST that allows for exact sinus up to 10 kHz. A priori, the tester offers global results for permeability *µ*_G_, power function *p*_G_(*t*) and total loss *P*_G_, as averaged over the entire sample material. But beyond that, a so-called Experimental Window (EW) offers additional information on local characteristics, as determined in a small central “key hole” region of defined magnetization. Here, a scanning adapter is mounted to study localized crystallographic features of the grain structure, as well as inhomogeneities, like failures, structure modifications, or specific technological treatment. Out of several types of sensor units a linear motor drive takes up a specific one within little manual effort. Already-developed sensor concepts concern the local tangential field, permeability, power, loss, and local widths of main domains and spike domains. The paper discusses several examples of analyses.

## 1. Introduction

Inspired by the key hole concept in micro-surgery [1], we developed a Magnetic Key Hole concept to study localized characteristics of soft magnetic laminations under defined global magnetization conditions. In surgery, diagnostics or treatments should be performed with the organ in situ, with minimal impact on normal functions. With this, treatments are even possible with a transcranial endoscope. By way of analogy, our new magnetic concept of Single Sheet Tester (SST) allows testing at minimum deviations from natural conditions, in situ and under normal field conditions.

With the above in mind, we developed the abovementioned concept of a Magnetic Key Hole (MKH) for non-destructive tests under exactly defined induction conditions. In conventional testing of soft magnetic crystalline or amorphous bands, *global* characteristics tend to be determined using rather large samples of 50 cm length [2,3,4], according to trends in standards [5,6]. This high magnetic path length favors homogeneous induction along the sample axis. Precise digital synthetization, in connection with “true field air flux compensation” [2], guarantees exact sinusoidal global waveform *B*_G_(*t*) during the period of magnetization.

In contrast to the above concept for global studies, for *local* parameters of material, small samples tend to be prepared with sizes of single centimeters (e.g., [7]). Precise magnetization of the experimental region tends to be impossible, due to non-representatively small geometric conditions.

In the course of our development of MKH, we aimed to prepare a review of the current state of testing with documentation of exact magnetization conditions. As a matter of fact, we could not identify a single publication. We attribute this to three reasons:

- Ensuring precise side-conditions for the study of a specific local effect would need additional specific sensors for control.
- Additional sensors complicate experimental arrangements in ways that are often not justified.
- In many cases, systematic errors of testing are acceptable for understanding the given effect undergoing testing.

The above means that it has to be checked individually to determine whether the application of MKH is justified and whether the involved approximations and neglections are acceptable for the given study problem.

## 2. Basic Concept of Magnetic Key Hole Technique

For the first time, the concept of MKH proposed in this study is aimed at the link between precise global magnetization and local investigation in a restricted experimental region that contains defined sources of inhomogeneity. The target is to allow detailed physical tests of arbitrary types, including local permeability, loss and magnetostriction. But studies of local domain configurations should also be possible, without sample treatments (like de-coating and polishing).

**Figure 1:**
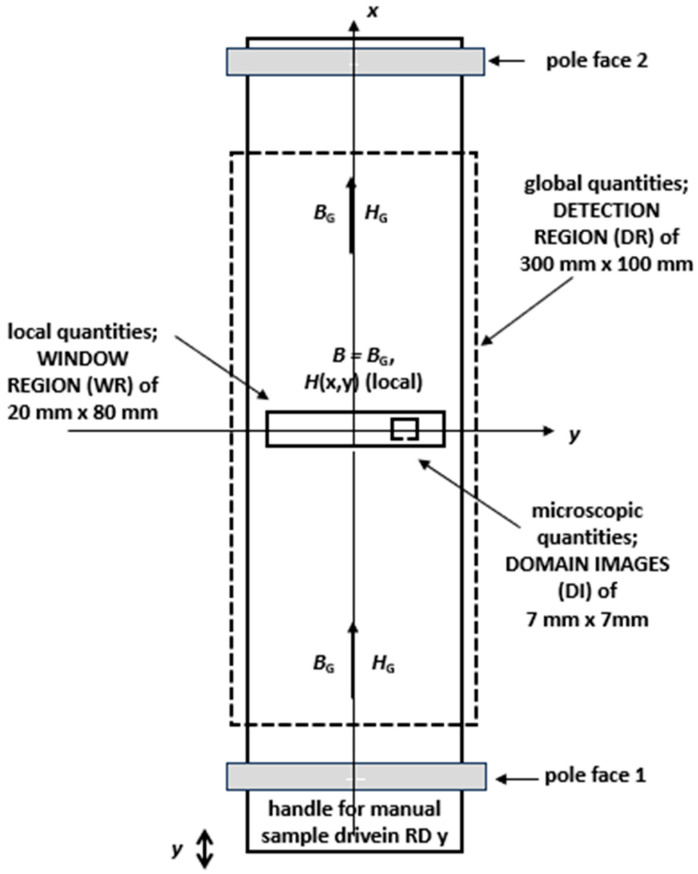
Assumption of three geometrical levels, G, L and M.

The above concept means that three geometrical levels are involved, as illustrated in Figure 1.

*A.*  ***The Global level G***

We define a *global* geometrical level G as the 30 cm long and 10 cm wide, so-called Detection Region (DR) of the investigated 50 cm long MF-SST sample. A width of 10 cm was chosen as a compromise between compactness and industrial representativity. In addition, 10 cm is under discussion for future SST standardization [6], already being fixed for AR [8]. Using the very precise magnetization synthetization of MF-SST [2], it is assumed that an exactly defined global induction vector ***B***_G_(*t*) is impressed all along the sample’s axis *x* in the rolling direction (RD).

In transverse direction (TD) *y*, we are aware that very weak regional induction components are present, e.g., from sample preparation or due to other impact factors, that cannot be prevented and thus are neglected. We even neglect y-components from defined sample irregularities of the central Window Region (WR), arguing that they should be studied by so-called Rotational Single Sheet Testers (RSST [9,10]) with sensors for both RD and TD.

In contrast, the present tester defines itself as a Single Sheet Tester (SST) with sensor elements that are restricted to the RD. We restrict with the convention that our system should show practice relevance, not just for basic studies, but even more for technical and industrial developments.

We assume constant global induction *B*_G_ all over the detection region in order to avoid vector complexity, as a source of theoretical complications. As already mentioned, 2D phenomena should be the subject of research by RSSTs, together with the complex aspects of specific sensing.

*B.*  ***The Local Level L***

We define a *local* geometric level L for the 20 mm long and 80 mm wide region of the Experimental Window (EW). Since this Window Region (WR) is the region of all our focused interest, we do not use an index for this local level, neither for field, nor for losses. Closer discussions are given further down.

*C.*  ***The Microscopic Level M of Domains***

Finally, for individual, specifically selected, square 7 mm “*microscopic*” geometrical sub-regions (frames), we define a level M for domain study configurations within the 2 cm × 8 cm Window Region (WR). By means of the colloid technique [11,12] or Faraday garnet technique [13,14], we perform analyses of main domains in such frames. For the case of GOES, we also consider spike domains. We interpret the results for individual grains (or grain complexes), introducing a specific coordinate *u* as the main direction [001] of easy magnetization, for theoretical interpretations.

## 3. Global Design of SST with Experimental Window

According to the above, the basic idea of EW is to integrate it into a magnetization device, for a sample with a geometry that is representative for usual types of soft magnetic lamination. This should consider both crystalline sheets like GOES and NOES, as well as amorphous ribbons (AR), in particular Fe-based ones.

In order to allow tests for all industrially important types of material, we developed first prototypes of EW for Multi-frequency Single Sheet Testers (MF-SST). As described in greater detail in recent publications (e.g., [2]), the testers are designed for a sample length of 50 cm. The sample width for EW is set to just 100 mm, in order to consider present concepts of standardization [6]. As an advantage, towards SST standard [5], the low width reduces the experimental expenditure, in particular for domain studies by colloid or Faraday technique.

**Figure 2:**
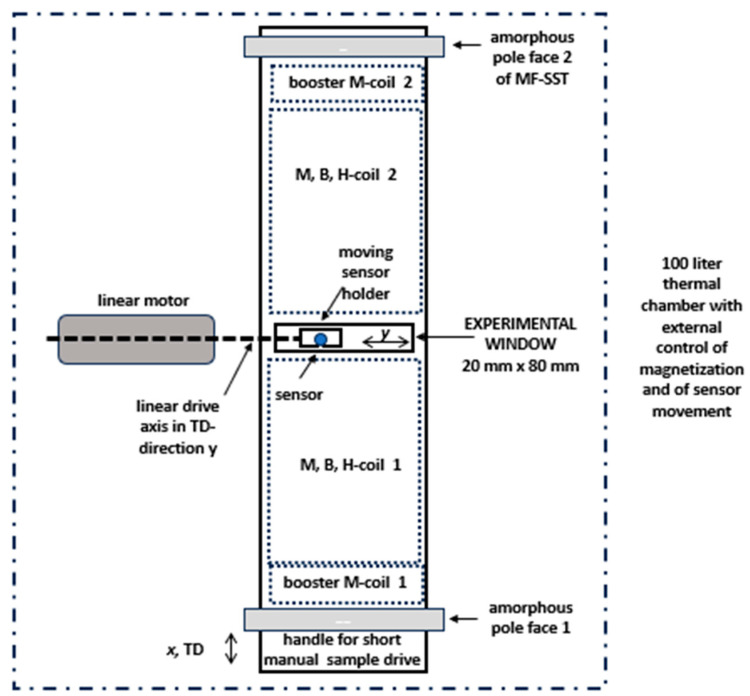
Schematic outline of the global practical design of EW in MF-SST, for a sample of 50 cm length and 10 cm width. In the simplest case, the scanning sensor (as depicted as a small grey circle) is given by a Hall plate for the detection of variations in the field *H* in y-direction, i.e., along the sample width.

The main challenge for EW design was to link controversial demands of non-affected conditions of magnetization with sufficient access for experimentally needed sensor components. This demand was fulfilled by restricting the key hole to a “window” of low length *L* = 20 mm in the x-direction, as illustrated in Figure 2. By doing so, the tester’s coil system (M-coil, B-coil, H-coil) exhibits a gap of just about 4% of length, with negligible impact on magnetization conditions for materials of high permeability.

In more detail, the window length *L* = 2 cm means that the magnetization coil (M-coil) of 300 mm was split into two portions of 140 mm each, in order to yield free access to the surface of magnetized sample. Further, the induction measurement coil (B-coil) was split. In practice, this is not critical, since the turn number used is as low as 40.

An important modification of SST concerned the H-coil for detecting the magnetic field strength of the sample surface [15,16]. With a total height of 1.6 mm—according to effective detection in 0.8 mm mean distance—the single coil of 300 mm length was split into two mechanically connected halves of 140 mm each. For defined positioning, a common layer was designed with a window of 20 mm length in RD and 80 mm width in TD (Figure 2).

According to the above, the basic idea of MKH was the alternative integration of different sensor types, for local tests of the 50 cm × 10 cm sample in its central region of 2 cm × 8 cm size. From its principle, the EW promises the application of sensor types like the following:

(a) Field sensors, like field plates, Hall plates, and tangential field coils;
(b) Induction sensors, as based on the needle method [17];
(c) Loss sensors, as based on a thermistor or on the electromagnetic principle;
(d) Domain sensors, as based on the Faraday effect or colloid (with a frame for normal field and for liquid concentration);
(e) Magnetostriction sensors (strain gauges, or optical; not yet tested).

**Figure 3:**
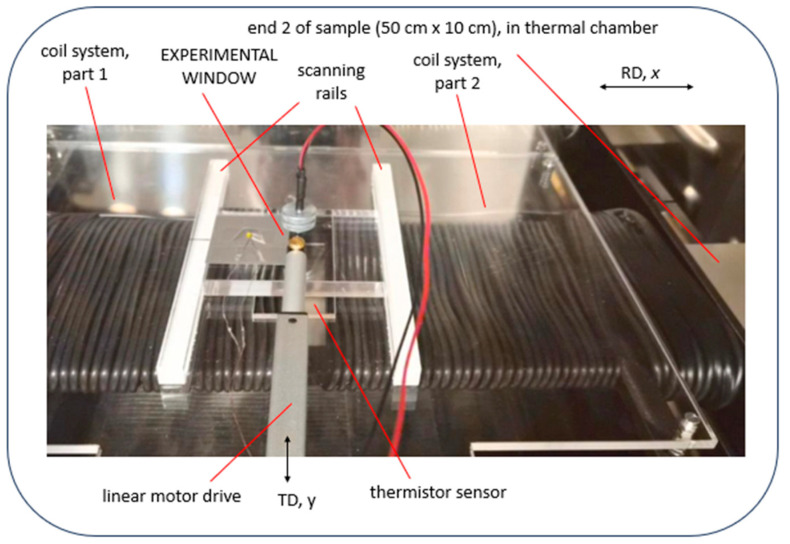
Photograph of tester with Experimental Window (EW), as arranged in a thermal chamber for calorimetric loss tests. A thermistor on rails can be moved in transverse direction (TD) by means of a linear motor.

As a global, practical design, the given sensor is mounted on a moveable sensor holder plate. As illustrated in Figure 2, the plate can be automatically moved in the samples transverse direction (TD), as the y-direction from about *y* = −30 mm up to *y* = +30 mm. The plate is guided by a rail system for precise positioning. Electric drive is based on a linear motor, with potentiometer for position control. This allows operation of the tester as arranged in a thermal chamber, for loss tests performed according to the calorimetric principle (Figure 3).

Position changes are also possible for the rolling direction (RD), from *x* = −10 mm up to +10 mm, by manual axial shift in the whole sample. According to Figure 2, the EW is not positioned in the exact center of the 50 cm long sample, but 2 cm shifted to the sample end, i.e., to position *x* = 27 cm. This shift offers the following two advantages:

(1) The sample front of ca. 2 cm length remains outside the apparatus and can be used as a handle for insert and pull-out of sample.
(2) Despite restricting its length to 2 cm, the EW allows for analyses along a 4 cm length via a second insert of sample in turned-around state—taking advantage of up to 2 cm shift. That is, the effective EW area is 4 cm × 8 cm = 32 cm^2^ instead of 16 cm^2^, which is significant, e.g., in cases of large grain sizes. Further tests can be performed for inverted sample, as being relevant for analyses of non-symmetric domain configurations, e.g., GOES.

In principle, the above concept allows for a semi-automatic scan of samples by exchanging different types of sensors. However, the main challenge was to perform controlled changes in sensor position—e.g., from a given grain A to a grain B—without opening the chamber door, to avoid affecting the thermal stability of the whole system of testing (Figure 3).

## 4. Local Analyses of Induction

**Figure 4:**
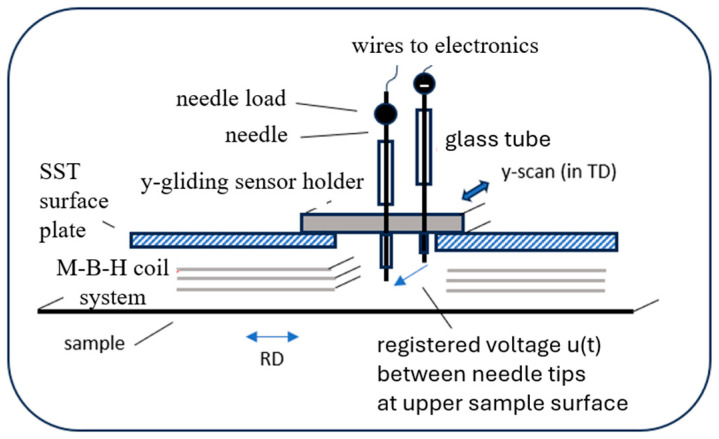
Conceivable schematic outline of the needle technique for analyses of local changes in the induction RD component *B* (note: lower parts of M-coils and B-coils are not depicted). A pair of two contact needles is vertically oriented in holes of a sensor holder + glass tubes. Needle loads favor good electric contacts to de-coated surface regions of the central EW sample region.

In principle, the application of the Experimental Window also comprises local analyses of induction. However, in practice, apart from very specific evaluations of domains (i.e., Bloch wall displacements), measurements are restricted to the use of the so-called needle technique [17,18]. Figure 4 shows a corresponding sensor system, for the detection of local changes in the longitudinal component *B* in RD. Already here, it should be stressed that we renounce tests in TD, due to high expenditure that is not justified considering the weakness of TD components.

As is well known, the technique uses two sharp needle contacts on two surface points A and B that yield a voltage

*u*(*t*) = *φ*_B_ − *φ*_A_ = *K* d*B*_G_/d*t*, (1)

with a constant *K* that considers several parameters. The voltage finally expresses the induction as averaged over the sample thickness. In technical practice, the voltage is 50% of an equivalent single-turn search coil, thus tending to be sufficiently high for routine measurements.

However, a precondition for effective measurements is good contacts between needle tips and the magnetic material. It means that a de-coated state of sample surface is needed which lacks scientific interest, at least for GOES, as the industrially most significant material. This is a reason to renounce the application of the needle technique within so far development of EW.

Due to the above, in our work, we have so far neglected local variations in induction in general ways, assuming homogeneous global induction, as determined by the global induction coils of the SST. This means that we set

*B*(*x*,*y*,*t*) ≈ *B*_G_(*t*). (2)

We justify the above approximation with the following arguments:

(a) EW-studies tend to be performed at considerably strongly magnetized soft magnetic samples at states with high permeability where inhomogeneities are characterized by strong percentage variations in magnetic field strength Δ*H* (in A/m), while the corresponding induction variations Δ*B* (in T) are very small.
(b) All studies are performed for global magnetization in RD, neglecting components in TD in a general way, avoiding the high corresponding experimental expenditure (leaving them to RSST). *B*_G_ is expected to arise all over the sample with weak local deviations, even in regions of defined material inhomogeneities.
(c) All analyses are of an approximate, rather qualitative nature, with the assumption that focusing on precision would be academic.

With the above, an experiment entitled as e.g., performed for 1.7 T as the global induction *B*_G_, tolerating small local deviations. Consequently, we use Equation (2) for evaluations of local values of permeability, but also for losses, as discussed in more detail further down.

Finally, it should be mentioned that the concept of EW actually *does* offer a possibility to estimate local induction values in regions of inhomogeneity. An effective way is to evaluate domain widening parameters as resulting from magnetization by means of domain analyses, as described further down in Section 6. As a significant advantage, both described methods can be applied on samples with a coating— also including scribing. However, the evaluated Bloch wall movements of course do not yield cross-section-averaged induction, but induction values of the observed sample surface.

## 5. Local Analyses of Field and Permeability

According to the above, analyses of local induction *B* are complicated by several impact factors. On the other hand, local variations in the sample’s surface field *H* can be performed with different types of sensors, in quite simple ways. Here we describe the application of a Hall plate [19] or a tangential field coil [20], respectively. They are simple tools for local analyses of local field variations, as caused by steel production, treatment of material, or by local defects of the given sample.

*A.*  ***Hall Plate Sensor System***

**Figure 5:**
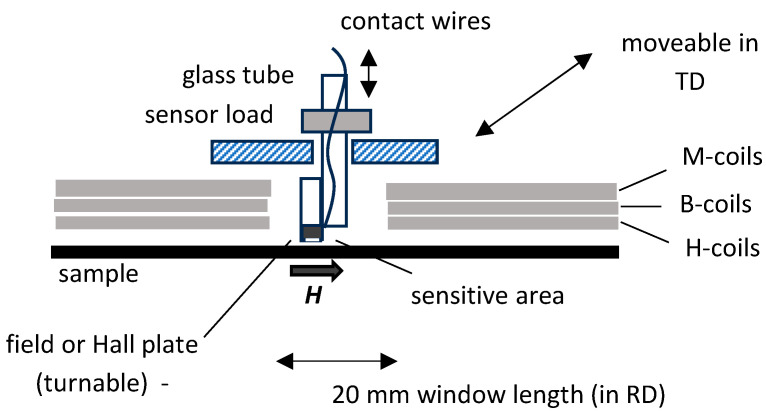
Schematic outline of plate sensor system in EW (note: lower parts of M-coils and B-coils are not depicted).

For field analyses within the EW, the a priori most attractive sensor type is given by a commercially offered Hall plate that yields a field-proportional voltage signal via straight-forward methods [19]. For analyses of local variations *H*(x,y) across the EW (Figure 5), the sensor can be moved by a linear drive in TD for up to about 80 mm. Manual movements are possible for axial sample shifts in RD. By rotating the sensor 90°, components in TD are also easily detectable.

**Figure 6:**
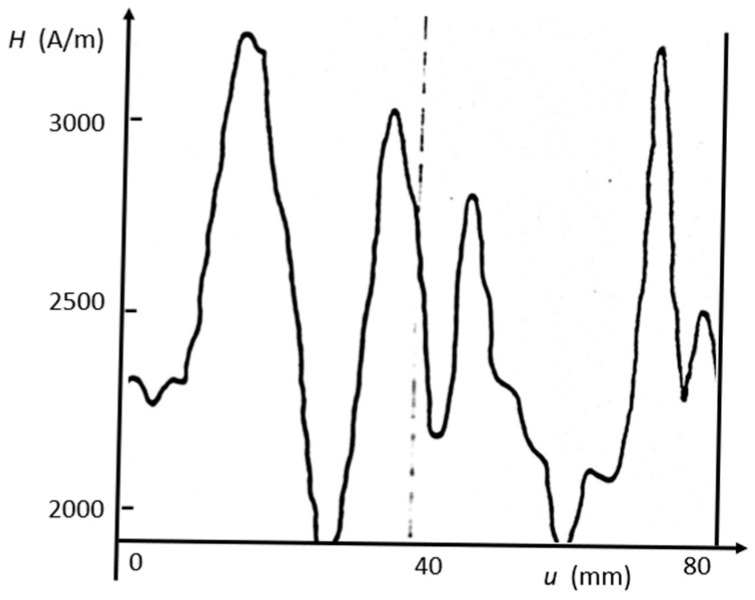
Example of result of Hall plate sensor, as applied for scanning of peak value *H*(s) of field in RD, for a sample of GOES, magnetized for a global induction *B*_GL_ = 1.7 T with *f* = 50 Hz.

With its small dimensions, the sensor can be used to detect inhomogeneities that can be related to the coarse grain structure of grain-oriented steel (GOES). Figure 6 depicts an example for field peak values in RD, as registered for AC magnetization of 1.7 T. We see field variations of an order of 50%, while the corresponding induction variations may be as weak as 2%. With this, we justify to assume *B* = *B*_G_ for local estimations of permeability and loss (see discussions further down).

*B.*  ***Tangential Field Coil System/Local Permeability***

An effective alternative to a Hall sensor proves to be to arrange a tangential field coil (H-coil) in the window [20,21]. As a basic advantage, the coil is a most simple two-wire element. However, it does not reflect the field *H* in a direct way, but its time derivative d*H*/d*t*. Thus, signal levels are weak for small values of magnetization frequency. Further, a high amount of wire turns tends to be necessary, except for very high values of *H*—apart from cases close to saturation or high frequency, respectively. This yields large sensor dimensions, in comparison to Hall plates.

**Figure 7:**
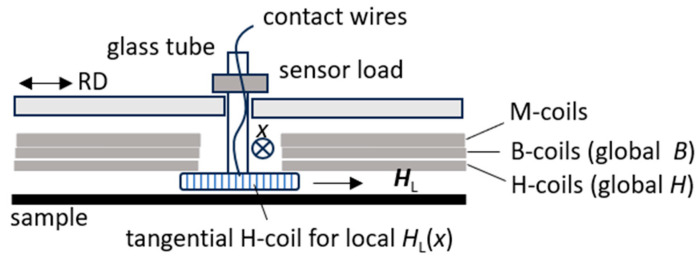
Schematic outline of tangential field sensor system (note: lower parts of M-coils and B-coils are not depicted).

As illustrated in Figure 7, the H-coil can be fixed at the end of glass tube in simple ways. However, contrary to a Hall plate it cannot be used for strongly localized analyses, due to needing many turns for sufficient sensitivity. Realistic minimum dimensions prove to be, e.g., 20 mm × 20 mm × 2 mm, corresponding to the EW-dimensions. Sufficient signal intensity follows, e.g., from *N* = 200 windings of 100 µm thick wire or of thin printed layers on a 2 mm thick plastic former. Tests for high induction also prove to be possible with a PCB-coil.

**Figure 8:**
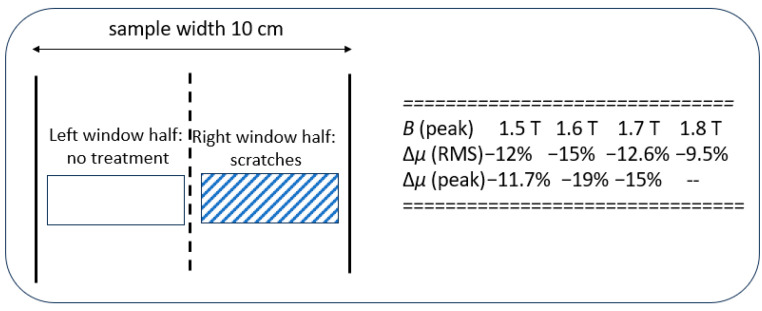
Consequences of diagonal surface scratches on the right window half of a sample of GOES, as resulting for local permeability (after [11]). Its peak value sinks by up to 20%.

As an example of application, Figure 8 shows the analysis of consequences of surface scratches on the stress coating of GOES. Analogous to laser scribing, mechanical scribing proved to be effective for domain refinements if applied in transverse direction (TD) [22]. On the other hand, here we arranged scribing in diagonal direction, in order to study artefacts with respect to decreases in permeability.

Exact determination of permeability would need a second moving sensor for local induction *B*(x,y)—e.g., induction needles. However, we assume that the corresponding experimental expenditure is not justified, due to the fact that the used SST generates global induction *B*_G_ that can be expected to arise all over the sample with weak local deviations, even in regions of material inhomogeneities. Thus, we assume

(2) *B*(*x*,*y*,*t*) ≈ *B*_G_(*t*),

and we define an effective permeability according to

*µ*(*x*,*y*) ≈ *B*_G_/[*µ*_o_ *H*(x,y)]. (3)

In principle, the measurement can be complemented by a second test procedure after a 90° turn of coil into the TD. However, this option would also not be justified, due to lacking practical (industrial) relevance, caused by small TD-components.

As an example, Figure 8 includes numerical results for permeability decreases as resulting form diagonal surface scratches applied by a ball pen on the right half of the window. As well known, horizontal scratches (in TD) tend to yield advantageous domain refinement. Diagonal ones would be preferable due to their weaker impact on the mechanical stability of the material. However, we see disadvantageous decreases in the material’s permeability, in particular for 1.6 T, as a frequent value for transformer cores.

## 6. Local Analyses of Energy Loss

So far, we developed two options for local analyses of loss. The first one uses the tangential field coil sensor, neglecting components in TD that tend to be very weak. As an advantage, this arrangement also allows the study of local instantaneous power for the first time. The second option uses the rise-in-temperature method, with the sample surface being scanned by the computer. Magnetization duration is up to 10 s which means that instantaneous studies are not feasible here.

*A.*  ***Electrodynamic Local Loss Analysis***

The electrodynamic loss measurement option is based on the loss formula that is usually applied for rotational single sheet testers (RSST) [10]:

*P* = 1/*ρ T* _T_∫ (*H*_RD_ d*B*_RD_/d*t* + *H*_TD_ d*B*_TD_/d*t*) d*t*, (4)

where the indices RD and TD consider two components of loss.

In principle, all above-mentioned options of field sensors can also be applied for local loss tests. However, local electrodynamic loss tests involve several specific problems:

(a) Exact tests would require also local determination of induction which however is not realistic, as outlined in Section 4. Setting global induction *B*_G_(*t*) yields test results that have approximate character a priori.
(b) The field has to be detected with avoidance of phase errors. For strong magnetization, even minute errors may yield strong errors of *P* which means that commercial Hall-meters are not effective.
(c) Applied specific Hall sensors need freely floating DC excitation which recommends the use of a shielded accumulator.
(d) Detection of field should be made in closest distance from the sample surface (<1 mm), in order to avoid impact of local changes in demagnetizing field components on the evaluated loss value.

**Figure 9:**
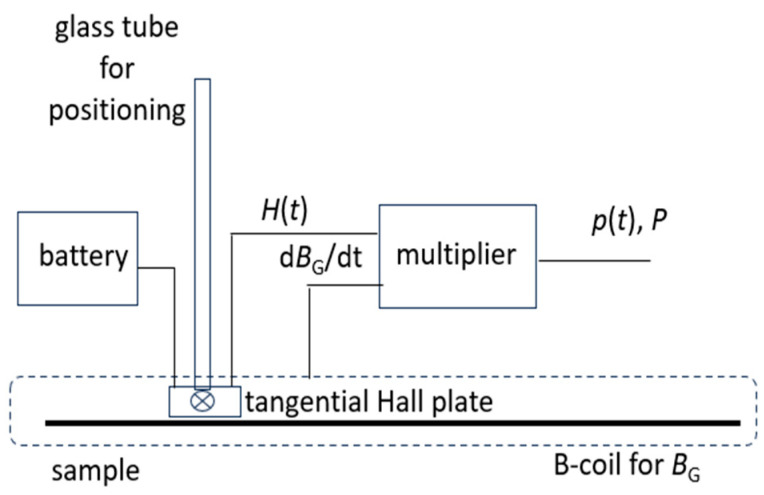
Schematic outline of tangential Hall sensor system as seen in axial RD-direction for approximate local loss determination. Within the EW, the Hall plate can be moved in TD in order to detect local changes in the sample’s surface field *H*, caused by the crystalline structure or by very small inhomogeneities due to, e.g., regional defects or technical treatments, respectively. For estimations of local loss, the local field *H*(*x*,*y*,*t*) is multiplied with the time derivative d*B*_G_/d*t* of global induction.

Figure 9 shows a corresponding experimental arrangement with a tangential Hall sensor of about 3 mm effective length and about 1 mm effective distance from the sample surface, excited by a 9 V battery. With the above, analogous to permeability *µ*, we restrict loss calculation to the simplified assumption

*P*(*x*,*y*) ≈ 1/*ρT* _T_∫ *H*(*x*,*y*,*t*) d*B*_G_/d*t* d*t* (5)

Apart from restricting to the detected field *H* in RD, here we also simplify matters by assuming that the local induction *B* within the window region does not deviate from the global induction *B*_G_. As in the case of permeability *µ*, we also here think that this is justified in order to avoid a procedure of academic nature. For example, studying local loss changes round a bolt hole of the sample, loss values are of technical interest for *B*_G_ rather than for exact local induction value *B*.

For loss tests in larger sample regions, we replace the Hall plate by a thin tangential field coil. In principle, the coil shown in Figure 7 for permeability tests can be applied also here. However, PCB-coils are unsuitable for loss determination due to the fact that according to

*P*(*x*,*y*) ≈ 1/*ρT* _T_∫ d*H*(*x*,*y*,*t*)/d*t B*_G_(*t*) d*t*, (6)

*P* for a given peak induction value depends on all H-values of the period, including very weak ones.

So far, we have applied the 30 mm wide, 20 mm long and 2 mm thick wired coil of *N* ≈ 200. Table 1 shows corresponding results for surface scratches analogous to Figure 8. Effects are similar to those for permeability, however, of weaker percentage intensity. As a tendency, loss decreases sink with rising induction *B*. On the other hand, they show weak increases with rising frequency *f*, probably due to the rising relevance of eddy current mechanism.

Here it should also be mentioned that Equation (5) or (6) also offers the possibility of calculating approximate values of local instantaneous power

*p*(*x*,*y*,*t*) ≈ 1/*ρT H*(*x*,*y*,*t*) d*B*_G_/d*t*. (7)

As more deeply discussed in [19], the following two instantaneous values are of particular practical interest:

A—The loss maximum *P*_MAX_: It tends to arise at instants where the highest amount of Bloch walls moves with maximum values of velocity, thus causing both the highest values of eddy current losses and highest values of hysteresis losses through interaction with pinning centers. This is mainly relevant for basic studies of loss mechanisms in the course of the assessment of novel steel materials under development. The maxima arise when the dynamic magnetization loop exhibits small instantaneous B-values.

B—The power peak values *p*+ and *p*−: In GOES, they tend to arise in instants of extreme field values. They express maxima of potential energy, as resulting from angular turns of atomic moments in direction of magnetizing field in RD, thus representing a measure of both crystalline anisotropy and misorientation.

Local determinations of the above characteristics at different grains within the EW promise insight on crystalline inhomogeneity of the studied type of steel, in particular with respect to different technologies of manufacturing.

*B.*  ***Rise in Heating as an Alternative Local Loss Test Method***

The basic mechanism of loss generation is the transfer of electromagnetic energy into heat. Thus, calorimetric measurement can be seen as the most straight-forward method for loss testing. In particular, this is true for local measurements within a narrow Experimental Window (EW).

From an experimental perspective, electrodynamic loss testing is perfectly suited for tests of large-area averaged loss *P* of a large sample (e.g., 50 cm × 10 cm). The induction *B* can be determined by a large B-coil, the field *H* by a large H-coil. For example, with 10 cm width and 10 cm length, sufficiently strong signals are detected for loss evaluation.

For local EW-tests, conditions are very different. In particular, electrodynamic tests at single grains are strongly complicated by small geometries, round 1 cm. In particular, both measurements of *B* and *H* are highly complicated if to be registered with phase control, as is needed for losses.

**Figure 10:**
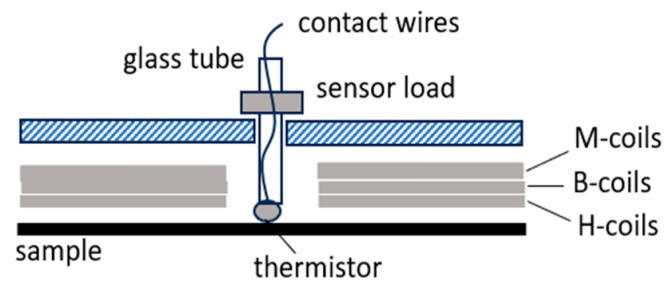
Schematic outline of sensor arrangement for calorimetric loss tests.

The above favors thermal tests by thermo-couples or by thermistors that have small dimensions a priori. Taking advantage of significant pre-work, we concentrate on thermistors of about 1 mm diameter that promise rapid local tests due to a low time constant *T*_c_ < 1 ms. Figure 10 shows a schematic of the currently used sensor system. The linear drive moves a plastic sensor holder for a sliding 2 mm thick glass tube with the thermistor at its lower end. For the defined press-on force on the sample surface, the sensor is loaded by a mass of 10 g.

For rapid local loss tests, we replace the conventional rise-in-temperature method with our novel rise-in-heating method, with the assumption that loss P does not depend on temperature at room temperature (18 to 30 °C). With this assumption, we magnetize the sample by a burst of induction *B*, e.g., for 1.7 T at 50 Hz. For loss evaluation, we assume that the corresponding peak value of heating expresses local loss according to

*P* = *k h*_P_, (8)

with *k* as a constant from empiric calibration, depending on many impact factors.

**Figure 11:**
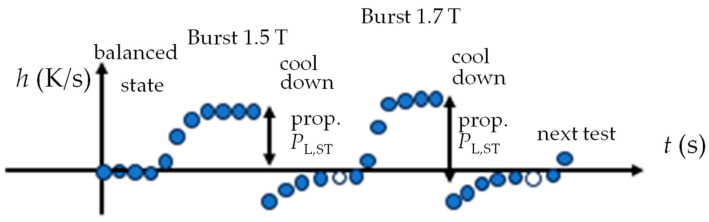
Basic principle of estimation of local loss P from the maximum of heating *h* as resulting from a magnetization burst of, e.g., 10 s duration (order of 1 µK/s for GOES magnetized with 50 Hz).

For simplicity, we restrict the test to a duration of 7 s, taking one measurement per second, as shown by the ball points in Figure 11. If started from balanced state (*h* = 0), *h*_P_ tends to reflect local loss in good approximation. But for a following test that is started after a short cooling time, it is necessary to consider the initial h-value. The most critical impact factor proves to be given by the high velocity *w* of heat transport along the area of material. We studied *w* by replacing a global B-burst by heating up a location A within the window with a hot soldering iron while monitoring *h* at a point B at 10 mm distance. The results indicate a velocity w of some mm, albeit mantled by the contacts A and B. We conclude that monitoring for a burst of 7 s should be evaluated from second 3 until second 7. This compromise provides a balance between sufficient evaluation time, local characteristics of result P and requisite cool-down time.

**Figure 12:**
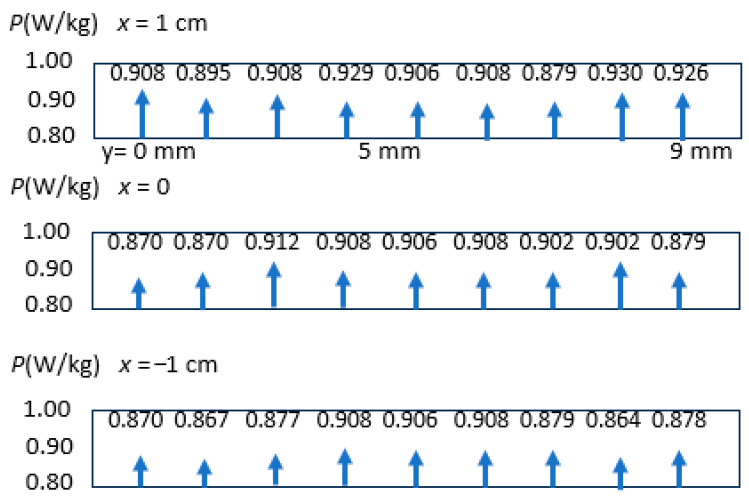
Example for loss variations as registered for 12 positions of a ca. 50 mm long region of GOES, magnetized with 50 Hz and 1.7 T (comprising about 8 individual grains).

For a demonstration of measured loss variations along the area of a sample of GOES, Figure 12 shows results of measurement for a global induction *B*_G_ = 1.7 T, for *f* = 50 Hz. The measurement was taken at a smaller sample with shorter B-bursts of just 7 s. The studied area of 20 mm × 90 mm shows loss variations of just 5%. It indicates that averaging through heat transport is still causing relatively balanced loss profiles. A better resolution of local loss extrema would need even smaller thermistor time constants, including contributions of even thinner glass tube holders.

As a main conclusion, a calorimetric study of crystallographically caused loss variations is highly demanding. On the other hand, thermic testing is assumed to be effective for tasks like the following:

- Regional comparison tests, i.e., relating sample halves/regions to each other, homogeneous/failure-affected to each other;
- Relating loss for extraordinary magnetization conditions (e.g., approaching saturation) to loss of well studied conditions to each other, or
- Studying effects of strong distortion (e.g., triangle or rectangular induction, vs. sinusoidal);
- Studying losses at very high frequencies characterized by excess *P* a priori.

## 7. Local Domain Analyses

So far, we have developed two options for local MKH analyses of magnetic domains. The first one uses the so-called colloid destabilization method. Compared to the Bitter method, it has the advantage that it can be used on samples with a coating, through application of a wetting agent. Coated samples can also be analyzed by the second option. It uses a Faraday garnet camera that scans the window region in order to take domain images for 7 mm × 7 mm sub-regions.

*A.*  ***Colloid Destabilization Method (CDM) With Normal Field***

We had already reported the basic principle of CDM much earlier in [12], and also then for analyses of GOES with domain refinement through stress coating and/or laser scribing. Actually, it is the simplest method for such analyses without requiring any apparatus. Destabilized colloid particles tend to be rinsed away by sample tilting. Since this is complicated by our narrow EW, we support clear domain contrasts by additional application of a weak static field in the off-plane direction.

**Figure 13:**
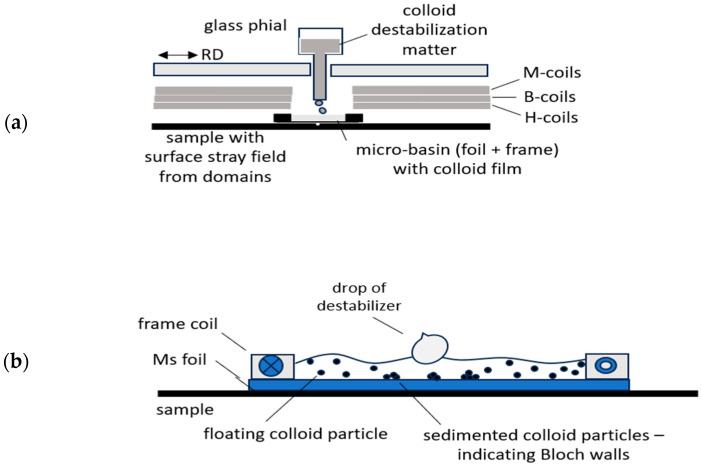
Schematic outline of sensor arrangement for domain images from the combination of colloid destabilization method (CDM) and off-plane field impressed by b frame coil. (**a**) Sensor arrangement. (**b**) Detail of micro-basin.

Considering the above, an effective experimental arrangement results as shown in Figure 13. Within the EW, we place a colloid basin of, e.g., 20 mm width and 60 mm length on the sample. Its bottom is given, e.g., by a 150 µm thick Al foil that does not clearly reduce the domain’s stray fields. On the foil, a frame coil is agglutinated as a container of about 1 mm deep water-based Ferrite colloid. The rectangular coil acts for both the wall of liquid and the wire for the magnetic off-plane field.

**Figure 14:**
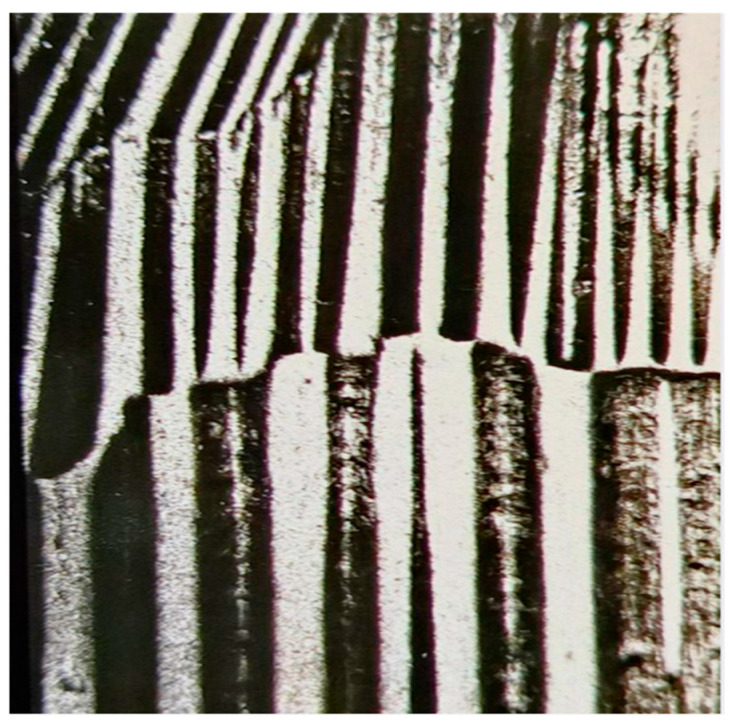
Colloid destabilization image of domains of coated GOES.

**Figure 15:**
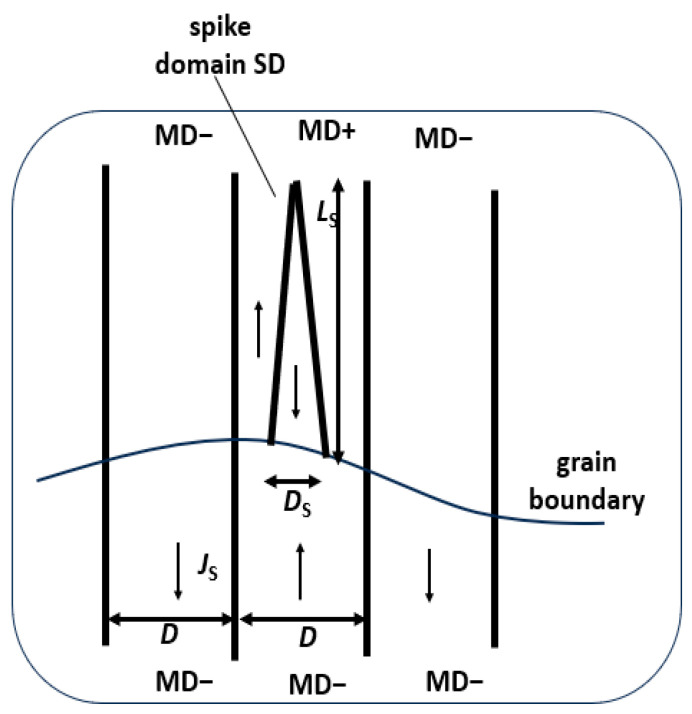
Concept for evaluation of registered domains of coated GOES.

After defined magnetization of the sample, the resulting local domain configurations of a selected sub-region of, e.g., 5 mm × 5 mm size can easily be camera-observed by pouring a drop of wetting agent (best option: ox gall, according to [12]) from the motor-moved eprouvette. Figure 14 shows a typical image and the Figure 15 the corresponding concept for evaluation of registered domains. Due to the off-plane field, it does not reveal Bloch wall positions, but domain faces, similar to images from Kerr effect or Faraday effect. We see a well-ordered constellation of wide main domains, with Bloch walls that pass over grain boundaries. Actually, the images coincide almost completely with those described in the following section for a completely different experimental procedure.

*B.*  ***Local Domain Analysis by Faraday Garnet Sensor***

**Figure 16:**
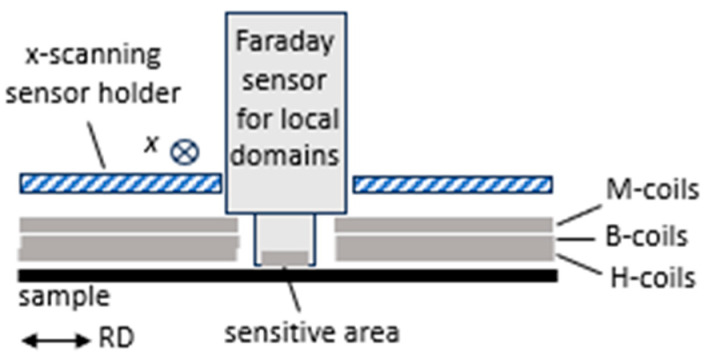
Schematic outline of sensor arrangement for domain images from Faraday garnet sensor system, mounted slightly above the sample surface to avoid damaging sensor face or sample, respectively.

As the second option for local domain analyses, we developed a moving Faraday garnet sensor system, as also described in [23]. Figure 16 shows a schematic outline of the system. A Faraday magnet camera (MagEye, Matesy) is mounted on the MKH-sensor holder for approximate zero distance from the scanned sample region. With a sensitive area of 7 mm × 7 mm, it offers about ten local images along the length of EW. As is well known, the contrast mechanism is based on the magnetization of the thin garnet foil, through the stray field of the samples’ surface magnetization patterns. This means that results can be directly compared with those of the above reported CDM.

**Figure 17:**
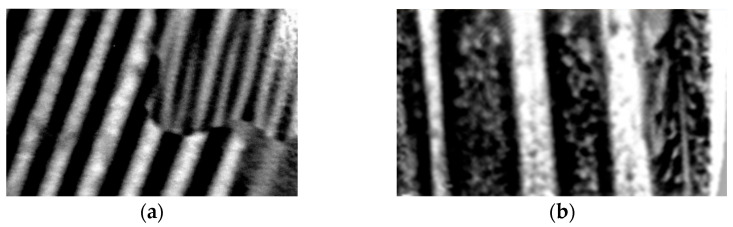
Examples of Faraday image of GOES. (**a**) Demagnetized state of main domains, the image including a smaller grain with spikes. (**b**) A sample magnetized for about 0.7 T, partly including extremely narrow long spikes.

Figure 17 shows examples for images of coated GOES. In comparison with Figure 14, this fully confirms colloid results, albeit with much lower experimental exhibitions. A priori, the optical technique yielded much weaker contrast. The latter proved to be significantly improvable by means of digital image processing, analogous to well-known topology-subtraction methods. This yields images similar to CDM with very similar optical features, identical contrast and identical resolution.

Figure 17 shows examples of very promising first results from samples of GOES, as attained in completely non-destructive way, without de-coating. For the demagnetized state, Figure 17a reveals a large grain with 14 main domains of about 500 µm width. As a specific finding, they are systematically coupled with main Bloch walls of a neighboring smaller grain of obviously higher off-plane mis-orientation angle *ß*. As it proves to be typical for the new materials, the latter causes generation of spike domains [22,24,25], as maintained even with magnetization (Figure 17b). The first results indicate that narrow spikes of width *w*_S_ ≈ *w*/2 and high length *L*_S_ of several mm (compare with the model in Figure 14) correlate with loss decreases through scratches, according to Table 1.

As described in detail in a separate publication, our present work reveals that such spikes as also described in may play an unexpectedly high, major role for the magnetization of GOES. At present, we are studying the consequences on losses by correlation of local domain images with local calorimetric results at specifically selected locations of the Experimental Window.

## 8. Discussion

This paper concerns experimental analyses of soft magnetic ribbons, including both electric steel and amorphous Fe-based ribbons. For such analyses, we can traditionally distinguish two types:

I Global studies—Global characteristics, such as permeability or losses, are investigated under exact definition of magnetization (induction, frequency, course of time, Form factor, etc.). Usually, the latter are guaranteed by a Single Sheet Tester (SST) with a large sample of 50 cm length.

II Local studies—This includes specific behaviors, such as arising from crystallographic inhomogeneities, or from local material defects. They tend to be investigated by specific sensors, on rather small samples of cm dimensions, under magnetization conditions of approximate physical definition.

As a completely novel approach, we put forward a so-called Magnetic Key Hole (MKH) method for discussion, the aim of which is to combine the two types of analysis:

ad I—A large material sample measuring 50 cm length and 10 cm width is magnetized by SST under the best defined conditions. In particular, the global magnetization is optimized by synthesized excitation, supported by both an amorphous yoke and two booster coils. Global *µ* and *P* are determined by extra-large B-coils and tangential H-coils, for a sample region of at least 30 cm length and 10 cm width, i.e., ca. 300 cm^2^ area.

ad II—Concentrated towards the center, local analyses are performed in a Magnetic Key Hole (MKH) of 2 cm × 8 cm area, i.e., 16 cm^2^, yielding a portion of about 5 percent. With just a 3 cm length, the global magnetization remains almost unaffected. However, electrically controlled scanning with local sensors facilitates effective analyses of characteristic inhomogeneities. They may result from the manufacturing of material, like grain structures. But they may also be technically introduced, e.g., by stress-coating or scribing of electric steel. Finally, defects like stacking holes may also be studied, with attempts to ensure minimum impact on technical performance of the soft-magnetic device.

In practice, the above concept yielded two major problems of design:

Problem I—For ideal conditions of magnetization of the 50 cm long material sample, a modern SST applies a coil system of up to about 40 cm length that has to be split in its center without significant impact on global magnetization. This demand proved to restrict the gap length to approximately 2 cm as the useable length of the “Experimental Window” (EW). This complicated the global design of apparatus, however, with acceptable expenditure.

Problem II—For effective experiments within the EW, different sensors had to be designed with small dimensions. This proved to be simple with respect to local field *H*, applying Hall plates or tangential field coils above the sample. But it proved to be crucial with respect to induction *B* where drilling of holes for local B-coils is not attractive owing to their destructive characteristics. The alternative to using surface needles was unattractive due to lacking space in the rolling direction (RD). Thus, measurement of local *B* had to be replaced by assuming global *B*_G_ in approximation. Due to high values of permeability *µ*, this compromise proved to be acceptable for the estimation of local µ-values. However, for local losses *P*, it represents a significant source of error. As a solution, the whole tester had to be operated in a thermal chamber, in order to allow calorimetric loss tests at rather high levels of expenditure.

With the above compromise, many quantitative results of EW are of approximative nature. However, this is acceptable for studying the consequences of local inhomogeneities or defects in the material, since they lack defined conditions a priori. In particular, this is valid for analyses on the microscopic level of magnetic domains. Such analyses were complicated by the need to perform the study in the thermal chamber. However, this problematic was satisfied by design of automatic control of sensor location from outside, at least in transverse direction (TD), while small shifts in RD were offered in manual ways.

With the above compromises, the novel concept of Experimental Window offers a completely new test system for the link of global magnetic measurements of high precision with local analyses of at least qualitative relevance. The system combines tests on global level G with those on local level L, or even (restricted) microscopic level M.

Table 2 summarizes the applicability of the MF-SST with EW for dimensions that range from about 500 mm down to 0.1 mm. The value 500 mm concerns the total length of the investigated soft magnetic sample which has a width of 100 mm. At the global level, the apparatus exhibits a pair of 150 mm long B-coils that determine the global induction *B*_G_(*t*). A pair of H-coils are mounted for the corresponding field *H*_G_(*t*). The signals yield the power function *p*_G_(*t*) and the loss *P*_G_ as its time average. This information offers an effective reference for many local analyses.

The latter concerns sensors at the local level in the EW. Here it should be repeated that the EW is designed for the alternate mounting of a single sensor on the moto-driven sensor holder. Exchanging the sensor type proves to be possible typically within 15 min which tends to be acceptable. An exception concerns the thermistor sensor, where stabilization times of one hour may be necessary. On the other hand, rapid tests of local field *H*(*x,y,t*) are available using H-coil or Hall-plate. Non-thermal tests of local loss are available by linking the local signal *H*(*x,y,t*) with the global signal *B*_G_(*t*), while assuming homogeneous induction and neglecting components in TD.

With respect to domains, the colloid technique has so far not been applied within the complete apparatus. However, extensive prior research predicts feasibility, apart from restrictions to attain full rinse-off of superfluous colloid, as described in [12]. On the other hand, stable mounting of a Faraday sensor guarantees effective images in combination with existing software contrast enhancement techniques.

Indeed, the concept shows clear analogies with medical applications of Key Hole Techniques for local performance of inspections, diagnoses and surgical operations. Further, future improvements may concern quantitative developments of even smaller sensors, including sensors for local components in the transverse direction.

## 9. Main Conclusions

The concept of Magnetic Key Hole (MKH) with Experimental Window (EW) proposed here is aimed at the link between precise global magnetization and local investigation in a restricted experimental region that contains sources of inhomogeneity. For example, a left sample half of ideal state is compared with sample failures in the right sample half. Consequences of failures can be studied on three levels: the global level G, the local level L within the window, and the microscopic domain level M in the sub-region of the window. The target is to facilitate detailed physical tests of arbitrary type, with alternating arrangements of a suitable sensor, e.g., for local permeability, loss or magnetostriction. But studies of local domain configurations are also possible, including tests in a magnetized state. The above options yield a completely novel sensor system for multi-parametric studies of crystalline or amorphous ribbons, as magnetized by modified MF-SST.

## Figures and Tables

**Table 1:** Consequences of diagonal surface scratches on a sample of GOES, as resulting for local loss *P*.

*B* (Peak)	1.0 T	1.3 T	1.5 T	1.6 T	1.7 T
Δ*P* (50 Hz)	−9%	--	−6.4%	−6.4%	−6.9%
Δ*P* (100 Hz)	−9%	−8.8%	−8.4%	−8.9%	−7.4%
Δ*P* (200 Hz)	−9.2%	−9.5%	−8.3%	−6.7%	−5.6%

**Table 2:** Survey of dimensions of SST with Experimental Window (EW).

Levels	Dimensions	Sensors (Examples)	Targets
global G	300 mm 100 mm	H-coil (300 mm × 100 mm) B-coil (300 mm × 100 mm)	global field *H*_G_ global induction *B*_G_ global power *p*_G_, loss *P*_G_
local L	30 mm 10 mm	small H-coil (30 mm × 30 mm) B-needle tips (30 mm × 30 mm) thermistor (1 mm, 10 mm averaging)	local field *H* local induction *B* local loss *P*
micro M (domains)	3 mm	tangential Hall plate (3 mm × 1 mm) colloid basin (10 mm × 30 mm) Faraday sensor (7 mm × 7 mm)	local power *p*, loss *P* grain sizes domain
	1 mm		configurations main domain widths *d*
	0.3 mm		spike domains (widths *d_s_*, lengths *L_s_*)

## Data Availability

The original contributions presented in this study are included in the article. Further inquiries can be directed to the corresponding author.

## Abbreviations

AR		Amorphous Ribbon (considering Fe-based)
CDM		Colloid Destabilization Method
DR		Detection Region, of 30 cm length and 10 cm width
EW		Experimental Window, of 2 cm length and 8 cm width
GOES		Grain-Oriented Electric Steel
MKH		Magnetic Key Hole Technique
NOES		Non-Oriented Electric Steel
RSST		Rotational Single Sheet Tester
RD		Rolling Direction (in *x*)
SST		Single Sheet Tester
TD		Transverse Direction (in *y*)
WR		Window Region (“key hole” of 2 cm × 8 cm)
*B*_G_	(T)	induction, global of DR
B(x,y)	(T)	induction, local within WR; mostly assuming *B* = *B*_G_
*B*_M_(*x*,*y*)	(T)	induction, local within WR; “microscopic” value as determined from main domain widenings *w*
D	(µm)	main domain width
*D*_S_	(µm)	spike domain width
*H*_G_	(A/m)	magnetic field strength, global of DR
*H*(*x*,*y*)	(A/m)	magnetic field strength, local within WR
*h*(*x,y*)	(K/s)	heating (rise in temperature), local within WR
*J*_S_	(T)	spontaneous polarization (assuming 2 T for GOES)
*L*_S_	(mm)	spike domain length
*P*_G_	(W/kg)	losses, global of DR
*P*(*x*,*y*)	(W/kg)	losses, local within WR
*p*_G_(*t*)	(W/kg)	instantaneous power function, global of DR
*p*(*x*,*y*,*t*)	(W/kg)	instantaneous power function, local within WR
*u*	(V)	voltage
***v***	(mm)	easy direction (ED); crystal-oriented
*w*	(mm/s)	heat transfer velocity
*x*	(mm)	rolling direction (RD); axial
*y*	(mm)	transverse direction (TD)
*z*	(µm)	normal direction (ND); off-plane
α	(°)	mis-orientation angle in-plane of [001] towards RD
ß	(°)	mis-orientation angle off-plane
*ρ*	(kg/m^3^)	density of material
